# Does a Lack of Awareness of Cycloid Psychosis Hamper Adequate Treatment for Patients Suffering From This Disorder? A Case Report

**DOI:** 10.3389/fpsyt.2020.561746

**Published:** 2020-11-12

**Authors:** Armand Hausmann, Julia Dehning, Michel Heil, Laurin Mauracher, Georg Kemmler, Heinz Grunze

**Affiliations:** ^1^Department Psychiatry, Psychotherapy and Psychosomatics, Medical University of Innsbruck, Innsbruck, Austria; ^2^Psychiatrie Schwäbisch Hall & Paracelsus Medical University Nuremberg, Nuremberg, Germany

**Keywords:** cyclicity, mood stabilizers, lithium, valproic acid, catatonia, cycloid psychosis, classification systems, case-report

## Abstract

Categorial systems of nosology are based on a cross-sectional enumeration of symptoms with a predefined cut-off, but hardly capture rapid fluctuations of manifestation nor longitudinal characteristics, e.g., cyclicity. Especially with disorders presenting with an admixture or frequent change of psychotic and affective symptoms, diagnostic specifity of the DSM and ICD diminishes. In those instances, alternative concepts as cycloid psychosis might display more accurately the very characteristics and course of a mental disorder and help to tailor individualized treatments. Karl Leonhard described three major subtypes of cycloid psychosis: anxiety–happiness psychosis, confusion psychosis, and motility psychosis, all showing a pleiomorphic symptom profile resembling intraphasic switching of poles. Here we present the case of a 59-year-old woman suffering from cycloid psychosis as defined by the criteria of Perris. Between 2013 and June 2019, the patient was admitted 35 times for compulsory treatment. A frequent change of diagnoses, ranging from adjustment disorder to complex PTSD, and from unipolar depression to “pseudoneurotic schizophrenia,” resembles the puzzling manifestations. Most of the time the patient was labeled as schizoaffective disorder despite never displaying clear psychotic core symptoms. Despite treatment with different antipsychotics including LAI the cumulative length of hospitalization increased steadily from 74 days in 2014 to 292 days in 2017. When reviewing the case in 2017 the longitudinal pattern of her disorder and the diverse acute manifestations were finally conceptualized as a cyclic on-off of an atypical psychosis. After starting lithium to pre-existing LAI antipsychotics and valproic acid, the number of days per year spent in inpatient care sharply dropped to 136 in 2018. We propose to reconsider cycloid psychosis as a useful clinical concept whose descriptive value, validity and utility for treatment decisions should be further evaluated. Lithium alone or in addition to valproic acid may act on cyclicity as a core symptom of cycloid psychosis as well as of bipolar disorder, even in the absence of major affective symptoms.

Crude classifications and false generalizations are the curse of organized life!

George Bernard Shaw (1856–1950)

## Background

Emil Kraepelin proposed to dichotomize psychiatric disorders with psychotic features into two major categories: dementia præcox, later coined as schizophrenia, and manic-depressive insanity, conditions that were later named bipolar disorder and major depression. Different from the present classification systems ICD-10 (1) and DSM5 (2), Kraepelin's dichotomy was not based only on cross-sectional symptoms, but also took the long-term course into consideration. Over the past century there had been many proposals how to categorize conditions that do not fully fit into either group, e.g., the ICD-10 F23 category of “acute and transient psychotic disorders” (ATPD). This category summarizes quite heterogenous disorders with a few common denominators: acute onset (within 2 weeks); complete remission (one to 3 months); polymorphic, schizophrenic or predominantly delusional syndromes, and an association with stressful life events. With the introduction of ICD-11 (3), the former ICD-10 category F23 will be narrowed to the polymorphic subtype (ICD 11 category 6A23.0) that refers to the French concept of bouffée délirante, originally coined and described by Valentin Magnan (1835–1916) (4), and Kleist's (5) and Leonhard's cycloid psychoses (6), featuring varied and rapidly shifting delusions, hallucinations, perceptual changes, agitation, perplexity and emotional turmoil. Subtypes with schizophrenic and predominantly delusional symptoms are reclassified into other categories of the renamed section “Schizophrenia or other primary psychotic disorders,” the delusional subtype (ICD.10 F23.3) be incorporated into the revised category “Delusional disorder” (ICD-11 6A24) and the present ICD-10 categories F 23.1 (Acute polymorphic psychotic disorder with symptoms of schizophrenia) and F 23.2 (Acute schizophrenia-like psychotic disorder) will be collapsed into “Schizophrenia or other primary psychotic disorders, unspecified” (6A2Z) (7). Given that these changes in classification are just around the corner, it appears timely to commemorate cycloid psychosis and possible treatment implications. As the cycloid psychosis concept is not explicitly included in standard international diagnostic manuals and therefore not codable for settlement with insurance companies, its implementation in clinical practice has been limited (8). However, in contrast to the limited construct validity of ICD and DSM disorders, phenotypes as described by Wernicke, Kleist and Leonhard, based on lifelong diachronic observations have good reliability and predictive and face validity (9).

This concept of cycloid psychosis was introduced at the beginning of the twentieth century by the German psychiatrist Karl Kleist (1879–1960). Based on clinical observations he considered a psychopathological entity distinct from Kraepelin's manic-depressive insanity and Bleuler's schizophrenia (5).

Pursuing the tradition of Kleist, Karl Leonhard (1904–1988) considered cycloid psychosis as one of three subtypes of his schizophrenia spectrum concept: (1) Cycloid psychoses, characterized by abrupt onset, severe polymorphous symptomatology in which opposite types of symptoms occur, and which show a complete recovery from each phase, (2) Non-systematic schizophrenias that follow a periodic course of bipolar symptomatology comprising different psychic functions, such as affect, thinking, and psychomotor function, with partial remission between the episodes, and (3) Systematic schizophrenias that show an early insidious onset and a well-defined symptomatology, with more pronounced residual symptoms (10, 11). Within the group of cycloid psychoses, he elaborated three overlapping subtypes, namely anxiety-elation (later also called anxiety-happiness) psychosis, confusion psychosis, and motility psychosis (6). Different from ATPD which concentrates on changes in mental processing (psychosis, delusions) and mood, Leonhard also integrated emotion (anxiety, happiness) and motility into his concept, with the overall common landmark feature “cyclicity.” For each of his subtypes Leonhard proposed alternating cycling between a restricted-inhibited phase and an expansive-excited phase. An attempt to operationalize Leonhard's criteria for cycloid psychosis was made by Bräunig but never tested in a larger sample. It was Carlo Perris (1928–2000) who then uncoupled cycloid psychosis from the schizophrenia spectrum; a step that appeared still too revolutionary for ICD 11 and DSM5 (2). He rather considered cycloid psychosis as an affective spectrum disorder with the consequence that it can be treated effectively with antidepressants and lithium (12). This view is, in part, also supported by the effectiveness of electroconvulsive treatment in cycloid psychosis (13). Perris also proposed a more unitary syndrome with operational diagnostic criteria (14, 15) (Table 1). Cycloid psychosis according to Perris and Brockington's criteria have a high discriminant validity: Applying a discriminant analysis to for cycloid psychosis in a mixed sample of psychotic patients, Peralta and Cuesta showed that the discriminant model correctly classified 94.7% of the patients. Among the cycloid symptoms confusion and mood swings had the highest discriminant power (16).

**Table 1:** Diagnostic criteria for cycloid psychosis according to Perris and Brockington (15).

1. An acute psychotic condition, not related to the administration or the misuse of any drug, or to brain injury, occurring for the first time in subjects aged 15–50 years.
2. The condition has a sudden onset with a rapid change from a state of health to a full-blown psychotic condition within a few hours or at the most a few days.
3. At least four of the following must be present: a. Confusion of some degree, mostly expressed as perplexity or puzzlement b. Mood-incongruent delusions of any kind, mostly with a persecutory content c. Hallucinatory experiences of any kind, often related to themes of death d. An overwhelming, frightening experience of anxiety, not bound to particular situations or circumstances (pan-anxiety) e. Deeper feelings of happiness or ecstasy, most often with a religious coloring f. Motility disturbances of an akinetic or hyperkinetic type, which are mostly expressional g. A particular concern with death h. Mood swings in the background, and so pronounced as to justify a diagnosis of affective disorder
4. There is no fixed combination of symptoms; in contrast, the symptoms may change frequently during an episode and show bipolar characteristics.

Table 2 depicts the evolution of the cycloid psychosis as a psychiatric diagnosis and its relation to other entities of the affective and schizophrenic spectrum.

**Table 2:** The evolution of the cycloid psychosis concept.

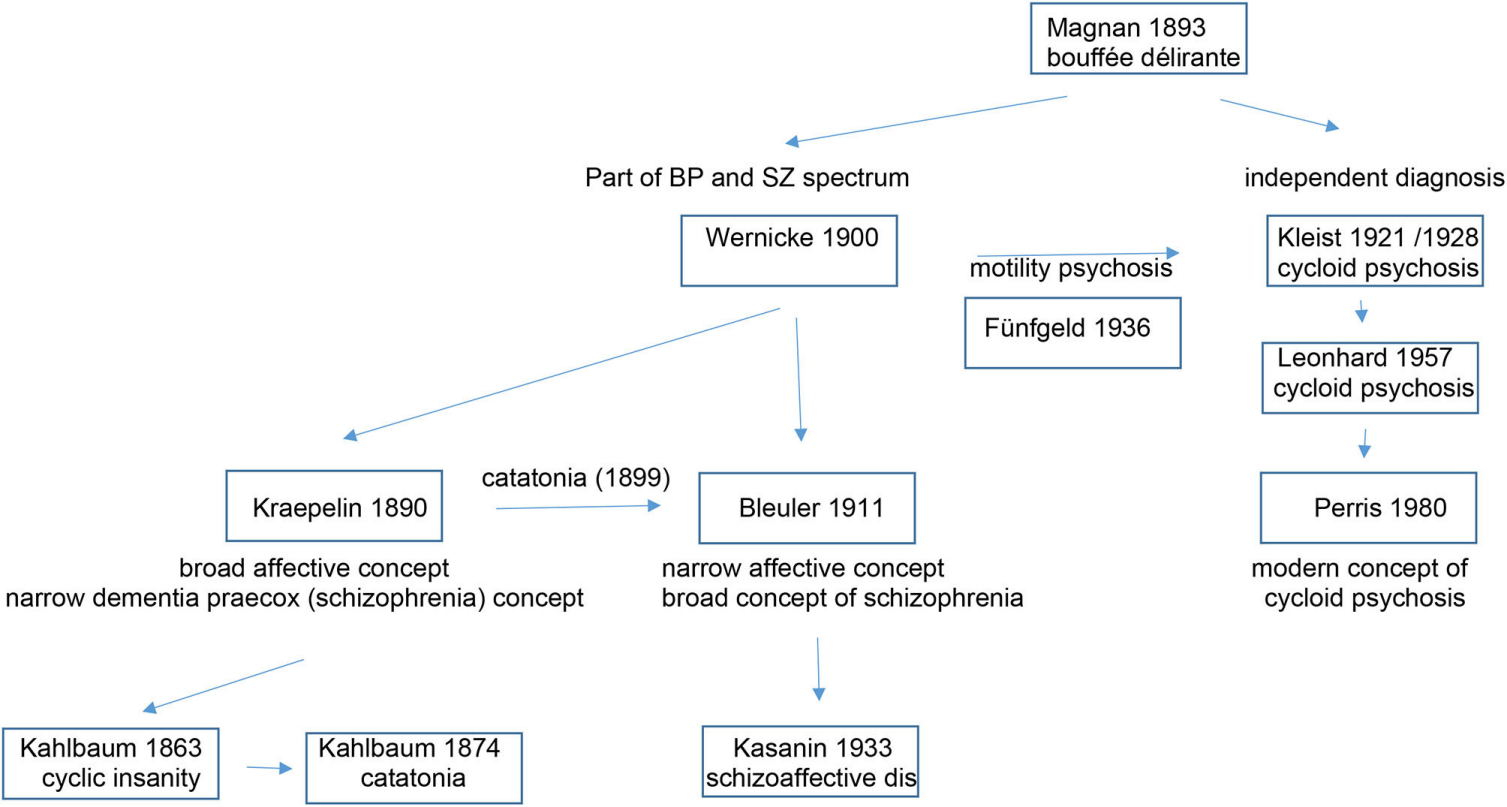

Cycloid psychosis as defined by the criteria of Perris is not rare: In a Swedish register study, the 1-year incidence for first admission with cycloid psychosis was 5.0 per 100,000 inhabitants in women and 3.6 per 100,000 inhabitants in men within the age range 15–50 years (17). Across epidemiological studies, and similar to what has been reported for ATPD (18), female preponderance has been a consistent feature (19). The impact of ATPD is significant, mortality rates are excessive and similar to those for bipolar disorder and schizophrenia (20). Reliable figures on mortality in cycloid psychosis are missing, but it appears fair to assume that cycloid psychosis and ATPD do not differ in this respect.

Cycloid psychosis appears not to fit either into strict categories of bipolar disorders (21) nor into schizophrenia or schizoaffective disorder (22) despite similarities with fuzzy boundaries (23). “Cyclicity” is usually considered as a core feature of bipolar disorder, and family studies have linked cycloid psychosis to bipolar disorder (30). Whether cycloid psychosis can be unequivocally delineated from bipolar disorder and schizophrenia, or has its place within a psychosis-affective disorder continuum (31) warrants further research. Studies on the pathophysiology of cycloid psychosis are still sparse, and some results are compatible both with a psychotic and a severe affective disorder (19). However, other studies also suggest neurobiological differences between cycloid psychosis, bipolar disorder and schizophrenia (24–27). In clinical routine, patients presenting with cycloid psychosis would be most likely assigned to DSM-5's unspecified psychosis category or brief psychotic disorders (19). The reason for choosing those soft categories is the cross-sectional plethora of diverse symptoms, including non-typical psychotic and affective symptoms, symptoms of confusion and other phenomena of motility, such as catatonic symptoms.

As catatonia is a prominent symptom of our case described in this article, we will elaborate a bit on its categorization. Catatonia is a psychomotor disorder that was subject to various descriptions reflecting the many changes in psychiatric disorder conceptualizations over time. In 1874, Karl Ludwig Kahlbaum (1828–1899) first described catatonia in his book “Die Katatonie” (28). Emil Kraepelin conceptualized catatonia as a manifestation of dementia praecox, together with hebephrenia and paranoia (29), and in 1908, Eugen Bleuler re-labeled dementia praecox to schizophrenia, with catatonia as one of its subtypes (30, 31). Despite its allocation to schizophrenia, pharmacotherapy with antipsychotic medications appears mostly ineffective (29). DSM5 now unchained catatonia from schizophrenia and reclassified catatonia into three diagnostic categories: catatonia associated with several different psychiatric diagnoses, catatonia associated with different medical conditions, and finally as an unspecified entity (2).

Here we present a case of cycloid motility psychosis intermittently displayed by a now 59-year-old woman suffering from a long-standing but until 2017 unrecognized cycloid psychosis. Complete records were available from March 2013 to April 2019. Primary outcome measure of the retrospective chart review was days per year spent in psychiatric hospital care. The patient gave written consent to publish her case while in a non-symptomatic interval of her disorder.

## Case Presentation

Starting with a first episode in 2013 aged 53y, shortly after losing her job as a child care worker, the patient was admitted for compulsory treatment 35 times until end of December 2019. Until her late forties, her psychiatric history was without peculiarities, birth and early development were normal. The patients' premorbid biography and life events are summarized in Table 3. Of note, the patient had a high level of functioning before the onset of psychosis in 2013. In the patient ‘s biography there is no clear evidence for a prodromal decline as we would expect in typical schizoaffective disorder or schizophrenia. However, her relatives reported subtle changes of behavior preceding her first admission. In the months before admission she often left home without letting anyone know. Once she was found completely drenched after erratically straying around the city. She has no history of drug or alcohol use disorder. EEG (2017) as well as MRI (2013 and 2015) did not detect any abnormalities. Thyroid function was always within the normal range.

**Table 3:** Biosketch.

**Age**	
0–9	Regular birth and postnatal period. Living with her mother who suffered from depression; father suffering from alcohol use disorder
9	Due to her mother's illness she was admitted to a boarding school
10	Suicide of her mother
10–18	Growing up in an SOS children's home; continuous conflicts with her carer
17–20	Domestic science school, finished with graduation
20	Marriage
29	Birth of a son
30	Certificate to access higher education
31	Study of educational sciences and graduation from university
36	Job as an educator working with children for a private organization
46	Promoted to Head of the team
48–52	Familiar problems. Husband lost money with gambling and his job. Son had school problems due to drugs and gambling
52	Patient had to support the whole family financially
53 (2013)	Mental “break-down,” Displaying first symptoms. First admission to an inpatient ward. Initial diagnoses: depressive adjustment disorder; anorexia nervosa

At first and at consecutive admissions she usually presented very agitated and exited, unable to communicate and in need of sedation. On five occasions she was brought in by the police wearing hand-cuffs. After receiving sedative medication (lorazepam 2mg i.v.) she often adopted an embryonic posture, grinding her teeth, with transient confusion, mutism and unresponsiveness to questions. This stuporous behavior tended to switch erratically into a pattern of motor agitation with walking around at random. When the confusional state had settled, she was able to remember but not to explain her stereotype and erratic moving around for days. While acutely ill she also displayed a flattened affect, which normalized with remission. The patient always remitted fully from these episodic symptoms, but in contrast to Leonhard's proposal, she did not regain the level of functioning needed to perform in her former job as an educator.

With a delay of several years and with already 10 different diagnoses on record she was diagnosed with cycloid psychosis in 2017. Earlier diagnoses resembled the puzzling manifestations and ranged from adjustment disorder to complex PTSD, and from unipolar depression to “pseudoneurotic schizophrenia.” Although she showed no core psychotic symptoms- except on one occasion when she felt threatened by people wearing black clothes- she was labeled schizoaffective disorder most of the time. Intermittent confusion and activation were attributed to recurrent psychosis or mania. However, a neuropsychological assessment [Eppendorfer Schizophrenie Inventar (ESI) (32), Rorschach (33)] performed in 2014 did not provide any indication for a schizophrenia-spectrum disorder. The SKID II semistructured interview did not reveal evidence for a personality disorder.

**Figure 1:**
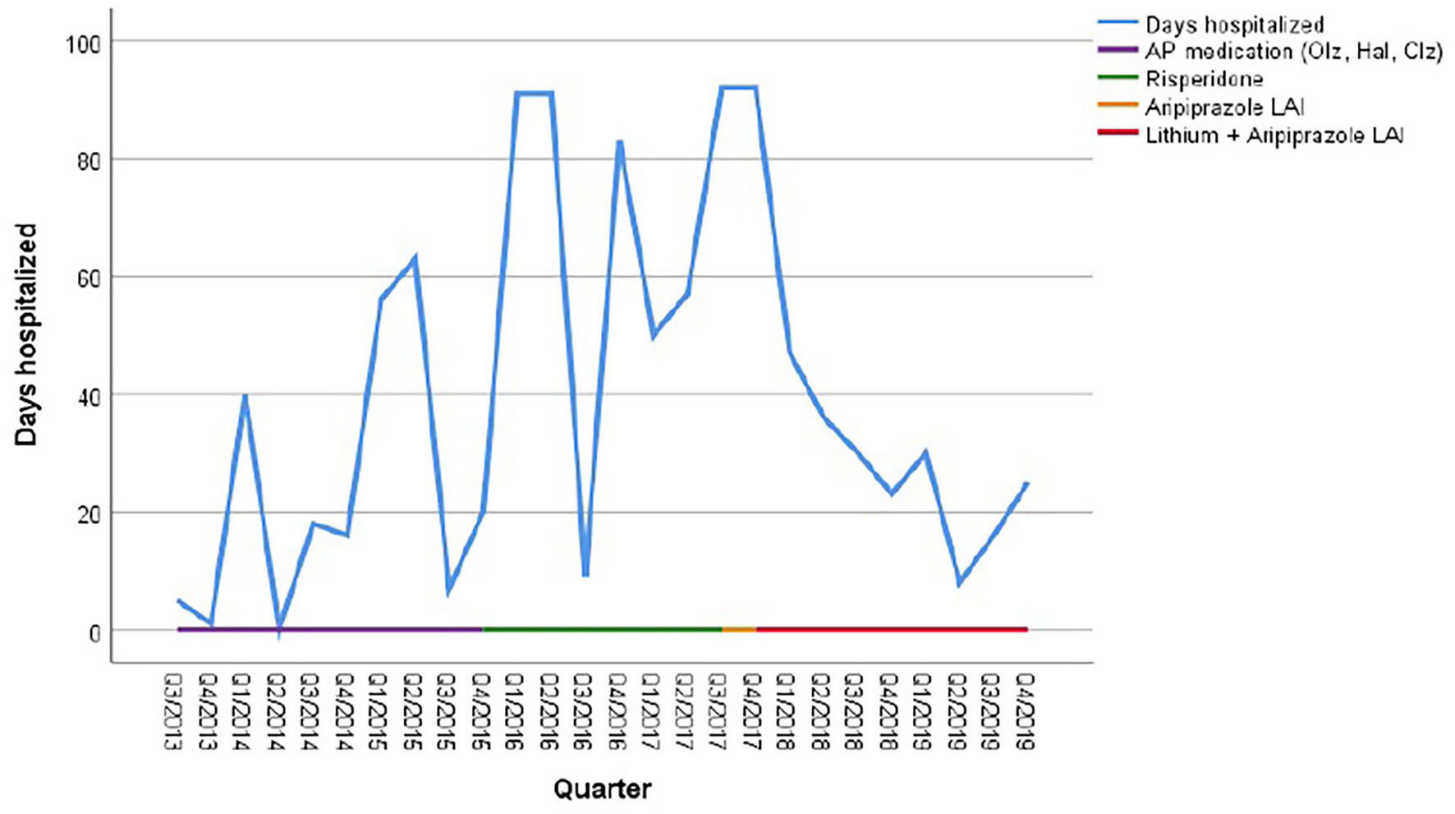
Cumulative duration of hospitalization in relation to psychotropic medication. AP, antipsychotics; Olz, olanzapine; Hal, haloperidol; Clz, Clozapine.

As illustrated in Figure 1 the patient received olanzapine (23.03.14–25.02.15) and haloperidol and haloperidol LAI (11.04.15–13.09.15) without any effect on recurrences. Clozapine was initiated on 10.12.15 but discontinued after 1 week due to marked side effects. From April 2015 to June 2017 she was treated with risperidone oral and LAI, which was then switched to aripiprazole. The cumulative length of inpatient stays, however, increased steadily from 74 days in 2014 to 292 days in 2017 (see Figure 1 and Table 4).

**Table 4:** Cumulative duration of hospitalization 2013–2019.

**Year**	**Days at risk**	**Days hospitalized (cumulative length of inpatient stays)**	**Percentage of the year spent in hospital**
2013	210	40	19.0
2014	365	74	20.3
2015	365	146	40.0
2016	366	274	74.9
2017	365	292	80.0
2018	365	136	37.3
2019	365	71	19.5
Total	2401	1033	43.0

In 2017, for the first time her symptoms were classified as a cyclic appearance of alternating hyper- and akinetic catatonia. Due to the cyclic fluctuation of motility, paired with confusion, and following the suggestions of Perris (12) and El-Mallakh (19), mood stabilizing treatment was initiated, initially with valproic acid 500 mg bid in addition to aripiprazole LAI. However, addition of valproate did not result in a reduction of recurrences. Therefore, lithium was added in November 2017 to the ongoing regimen with serum levels around 80 mmol/L. As a result, the number of days spent in hospital decreased to 136 days (or 37, 5% of the year) in 2018, whereas in 2017 the patient spent 80% of the year in hospital. During 2019, this percentage further decreased to 19.45% (Table 4).

In 2018 and 2019, the patient repeatedly discontinued the oral medication as she felt stigmatized by a psychiatric diagnose. At each reimplementation of this medication regimen, response appeared accelerated compared to previous years without lithium. Despite these relapses due to non-adherence, the number of days in hospital still came down to 71 in 2019.

## Discussion

Already Kahlbaum stressed the necessity of juxtaposing the transitory acute symptoms and look for periodic patterns underlying specific disorders. He postulated that the true nature of disorders will only reveal itself through their progression (32, 33).

After unsuccessful treatment with a variety of antipsychotics, it was decided to give less weight to the complex and varying acute symptomatology but focussing on the cyclic nature of the longitudinal course. The addition of lithium to valproic acid and aripiprazole LAI led to a decrease of the patient's hospitalization risk by nearly 50 % despite her low adherence, repeatedly stopping medication on average 3 months after discharge.

As the patient was admitted to four different hospitals and, within our hospital, to four different wards specialized in schizophrenia, in psychosomatic medicine and in affective disorders, changes in diagnostic classification might, in part, resemble also diverse diagnostic and clinical thinking. Independent of diagnosis, however, psychopharmacological treatment focussed on “psychotic” symptoms and not on cyclicity as the key feature. Antipsychotics were given throughout the observational period albeit with insufficient effects.

Cyclicity is a unifying feature of the different subtypes of cycloid psychosis according to Leonhard, and has also been described in other case reports, e.g., of anxiety-happiness cycloid psychosis (22) or excited-inhibited confusion psychosis (34).

Cyclic changes of motility were the key symptom in our patient. The following symptoms which, according to Kahlbaum (28), characterizes catatonia were observable: She displayed dysfunctional muscle tone and posture, such as rigor, stereotypical movements as well as psychomotor agitation. In addition, her interpersonal behavior fluctuated between mutism and aggressiveness.

Among others, the differential diagnosis of a psychotic bipolar disorder needs to be discussed. Psychomotor hyperactivity in acute mania can be difficult to differentiate from hyperkinetic motility psychosis. Changes in psychomotor activation levels are a core symptom of bipolar disorder, and catatonia might occur in acutely manic patients (35), more likely in delirious mania (36), but especially in adolescents (37).

Peralta and Cuesta (38) as well as Brockington et al. (39) analyzed index episode in relation to lifetime psychopathology, and the cycloid psychosis diagnoses emerged primarily in index episode ratings. Cycloid psychosis and bipolar disorder were difficult to differentiate and many patients diagnosed as cycloid at the index episode were later on diagnosed as individuals with atypical schizophrenia or schizoaffective disorder, bipolar type (38) which suggests that the cycloid psychosis concept may have less diagnostic stability in the long term. However, changing diagnostic habits and classification systems over time might also have biased the outcome. What might differentiate stable cycloid psychosis from bipolar disorder and especially schizophrenia in the long run is a more favorable clinical outcome, e.g., a lack of affective or behavioral defective states (40). According to a study by Jönsson et al., confusion symptoms appear to be prognostically favorable, whereas motility symptoms without confusion seem to indicate an unfavorable course of cycloid psychosis (41). On the epidemiological and symptomatic level, a large Danish registry study reports that acute onset, polymorphic symptoms, early remission, absence of premorbid dysfunctions and association with female gender are features that distinguish the narrow ATPD of ICD-11 (that largely covers manifestations of cycloid psychosis) from schizophrenia (42). In line with this, patients with cycloid psychosis typically present a premorbid adjustment similar or better to affective disorders (16) and much better than in patients suffering from schizophrenia (43). In a study by Peralta and Cuesta cycloid psychosis could be discriminated on a significant level on the basis of psychopathological symptoms either from schizophrenia or mood disorders, except of negative symptoms (16). The features examined included Psychotic dimension, Disorganization dimension, First -rank symptoms at index episode, First-rank symptoms lifetime, Depressive symptoms at index episode, Depressive symptoms lifetime, Depressive syndrome at index episode, Depressive syndrome lifetime, Manic symptoms at index episode, Manic symptoms lifetime, Manic syndrome at index episode, Manic syndrome lifetime, Psychotic/mood symptoms at index episode and Psychotic/mood symptoms lifetime. Preliminary results also suggest neurobiological differences between schizophrenia and cycloid psychosis. In cycloid psychosis, glycine levels appear elevated and tryptophan levels lowered as compared to schizophrenia (44) suggesting a more pronounced involvement of neurotransmitter systems other than the dopaminergic system.

However, in the absence of other manic core symptoms such as grandiosity, irritability or pressured speech we considered a bipolar diagnosis less likely.

Studies comparing cycloid psychosis according to Leonhard vs. non-cycloid psychosis or patients with schizophrenia suggest a better acute treatment effect of antipsychotics in cycloid psychosis patients (44, 45). Nevertheless, the cyclic changes of motility, despite presenting with a flat affect and lacking symptoms of anxiety or happiness, were at least partly responsive to lithium treatment. This might hint toward cycloid psychosis being a part of a broader bipolar spectrum disorder although family studies do not support this hypothesis (21). Alternatively, this case might suggest that lithium's beneficial effects in recurrent (cyclic) disorders is not dependent on mood involvement, but an effect on cyclicity itself. More recently, Geoffrey et al. described genuine effects on lithium on core clock genes expression *in vitro* (46). Previous reports on the use of lithium in cycloid psychosis also suggest efficacy in the prevention of recurrence, and, similar to bipolar disorder, higher intraerythrocyte/plasma lithium concentrations predict response (12, 47).

The differential diagnosis of schizophrenia or schizoaffective disorder appears less plausible as she only displayed transient paranoid symptoms at one occasion and no other Schneiderian first rank symptoms of schizophrenia. In addition, structured interviews and tests (ESI, Rohrschach) did not support a diagnosis of schizophrenia. The dynamics of the case are also not typical for schizophrenia. Patients suffering from schizophrenia or schizoaffective disorder mostly display a gradual decline of functioning over a prolonged period of time. In addition, the onset of the disorder is usually in the teens or twens. Our patient, however, had a hight level of functioning before the sudden onset of symptoms at age 53, which she did not regain in the long run. This is in contrast to Leonhard and Perris, but in line with more recent literature that reports residual defects depending on the length of follow-up in up to 17% of patients suffering from cycloid psychosis (40). The SKID II semistructured interview did not reveal evidence for a personality disorder in 2014. Nevertheless, the patient was diagnosed three times after 2014 with combined personality disorder or personality disorder NOS. Our interpretation of these clinical diagnoses is the helplessness of colleagues confronted with bizarre symptoms not clearly attributable to major affective or psychotic ICD categories, and non-response to standard medication.

## Conclusions

The syndrome our patient displayed cannot be squeezed into operationalized criteria of either schizophrenia, schizoaffective disorder or bipolar disorder. With the unquestionable need to classify disorders according to strict categorial systems for research, communication, statistics and reimbursement purposes the concept of cycloid psychoses appears to be buried in oblivion. Early recognition of cycloid psychosis has an important implication for the assessment, treatment, and effective management of recurrent confusional states with alterations of psychomotor activity and brief psychotic episodes. Cycloid psychosis entails a distinct prognosis and requires a tailored treatment.

Lithium alone or in addition to valproic acid may have ameliorating effects on cyclicity itself, even in the absence of affective symptoms which are more prominent in cyclic anxiety–happiness psychosis.

### Limitations

The validity of the cycloid psychosis concept still remains uncertain due to a lack of confirmative field studies. We therefore propose to consider this condition as a meaningful clinical category whose validity and utility for prognosis and treatment should be further evaluated. Albeit based on single case reports, including this one, and in the absence of high-level evidence, we suggest for the time being to consider lithium treatment of cycloid psychosis, even in the absence of prominent mood symptoms.

## Data Availability Statement

All datasets generated for this study are included in the article/supplementary material.

## Ethics Statement

According to the local IRB no IRB approval necessary for case-presentations. In a non-symptomatic interval of the patient's disorder she gave written consent to publish her case.

## Author Contributions

AH: treating physician, concept of paper, several re-editions, last edition of the work, and first submission. JD: gathering data and first draft. MH and LM: conjointly wrote the historical background. GK: responsible for descriptive statistics and tables and graphic presentations. HG: re-edited the manuscript several times, added references a well as some important viewpoints to the discussion, last edition conjointly with AH, and final submission. All coauthors amended the last draft.

## Conflict of Interest

The last three years AH received research support from Janssen-Cilag, Roche, as well as speaker honoraria from Lundbeck, Pfizer, Lannacher and Servier. HG received grants/research support, consulting fees and honoraria within the last three years from Gedeon Richter, Janssen-Cilag, Pfizer and Servier. The remaining authors declare that the research was conducted in the absence of any commercial or financial relationships that could be construed as a potential conflict of interest.
